# Diammonium Glycyrrhizinate Exerts Broad-Spectrum Antiviral Activity Against Human Coronaviruses by Interrupting Spike-Mediated Cellular Entry

**DOI:** 10.3390/ijms26136334

**Published:** 2025-06-30

**Authors:** Shuo Wu, Ge Yang, Kun Wang, Haiyan Yan, Huiqiang Wang, Xingqiong Li, Lijun Qiao, Mengyuan Wu, Ya Wang, Jian-Dong Jiang, Yuhuan Li

**Affiliations:** 1 CAMS Key Laboratory of Antiviral Drug Research, Beijing Key Laboratory of Technology and Application for Anti-Infective New Drugs Research and Development, NHC Key Laboratory of Biotechnology for Microbial Drugs, Institute of Medicinal Biotechnology, Chinese Academy of Medical Sciences and Peking Union Medical College, Beijing 100050, China; 2 State Key Laboratory of Bioactive Substances and Functions of Natural Medicines, Institute of Medicinal Biotechnology, Chinese Academy of Medical Sciences and Peking Union Medical College, Beijing 100050, China

**Keywords:** coronavirus, diammonium glycyrrhizinate, broad spectrum, spike-mediated cellular entry, RBD-binding activity

## Abstract

Glycyrrhizic acid (GA) and its derivatives have been reported to have potent pharmacological effects against viral infections, including SARS-CoV and SARS-CoV-2. However, their antiviral mechanisms against coronaviruses are not fully understood. In this study, we found that diammonium glycyrrhizinate (DG) can effectively reduce infections of several human coronaviruses, including HCoV-OC43, HCoV-229E, and SARS-CoV-2, as well as newly emerged variants, with EC_50_ values ranging from 115 to 391 μg/mL being recorded. Time-of-addition and pseudotype virus infection studies indicated that DG treatment dramatically inhibits the process of virus entry into cells. Furthermore, we demonstrated that DG broadly binds to the RBD of human coronaviruses, thereby blocking spike-mediated cellular entry, by using TR-FRET-based receptor-binding domain (RBD)-ACE2 interaction assay, capillary electrophoresis (CE), and surface plasmon resonance (SPR) assay. In support of this notion, studies of molecular docking and amino acid mutation showed that DG may directly bind to a conserved hydrophobic pocket of the RBD of coronaviruses. Importantly, intranasal administration of DG had a significant protective effect against viral infection in a HCoV-OC43 mouse model. Finally, we found that combinations of DG and other coronavirus inhibitors exhibited antiviral synergy. In summary, our studies strongly reveal that DG exerts broad-spectrum antiviral activity against human coronaviruses by interrupting spike-mediated cellular entry, demonstrating the pharmacological feasibility of using DG as a candidate for alternative treatment and prevention of coronavirus infection.

## 1. Introduction

Coronaviruses (CoVs) are a highly diverse family of positive-sense, single-stranded, enveloped, non-segmented RNA viruses [1]. The coronavirus disease 2019 (COVID-19) pandemic, caused by severe acute respiratory syndrome coronavirus 2 (SARS-CoV-2), has posed a public health threat worldwide in the past few years. Severe acute respiratory syndrome coronavirus (SARS-CoV) and Middle East respiratory syndrome coronavirus (MERS-CoV), which are transmitted from animals to humans, can induce severe respiratory diseases in afflicted individuals [2,3]. In contrast, common human coronaviruses, including 229E, OC43, NL63, and HKU1, constantly circulate in the human population and usually cause mild to moderate upper-respiratory-tract illnesses. Although vaccines and therapeutic drugs are available, it is still necessary to develop other prophylactics and therapeutics that can effectively inhibit SARS-CoV-2 variants, especially those with a broad spectrum of coronaviruses [4,5,6,7,8].

Glycyrrhizae Radix et Rhizoma (Chinese name: Gancao) is a traditional Chinese herb that is widely used to treat a variety of diseases [9]. Glycyrrhizic acid (GA), an important bioactive ingredient of Gancao, has been used clinically for more than 40 years as a drug to treat liver diseases, particularly chronic hepatitis [10,11,12]. GA also has potent pharmacological effects against viral infections [13,14,15], including human immunodeficiency virus (HIV), hepatitis A, B, and C viruses, herpes simplex type 1 (HSV-1), varicella zoster virus (VZV), Epstein–Barr virus (EBV), human cytomegalovirus, influenza virus, SARS-CoV, and SARS-CoV-2. Moreover, it has been reported that GA can effectively inhibit the replication of SARS-CoV-2 by targeting the spike protein [16,17]. Diammonium glycyrrhizinate (DG), a derivative of GA, is more stable, more soluble, and exhibits more significant bioactivities than GA. DG has been used for treating hepatitis in clinical practice [18]. In the present study, to determine whether DG exerts broad-spectrum antiviral activity against coronaviruses, we tested its antiviral activity against multiple human coronaviruses in vitro, including newly emerged variants of SARS-CoV-2. Further investigation of the underlying antiviral mechanism revealed that DG appears to bind to a conserved hydrophobic pocket of the receptor-binding domain (RBD) in the spike protein of coronaviruses to disturb spike-mediated cellular entry. In vivo studies demonstrated that administration of DG via the nasal route produced significant protective effects, highlighting its clinical potential. Our results thus strongly support the further development of DG as an antiviral candidate for use as an alternative medicine for treatment and prevention of coronavirus infection.

## 2. Results

### 2.1. Diammonium Glycyrrhizinate Potently Inhibits Replication of Multiple Coronaviruses

**Figure 1:**
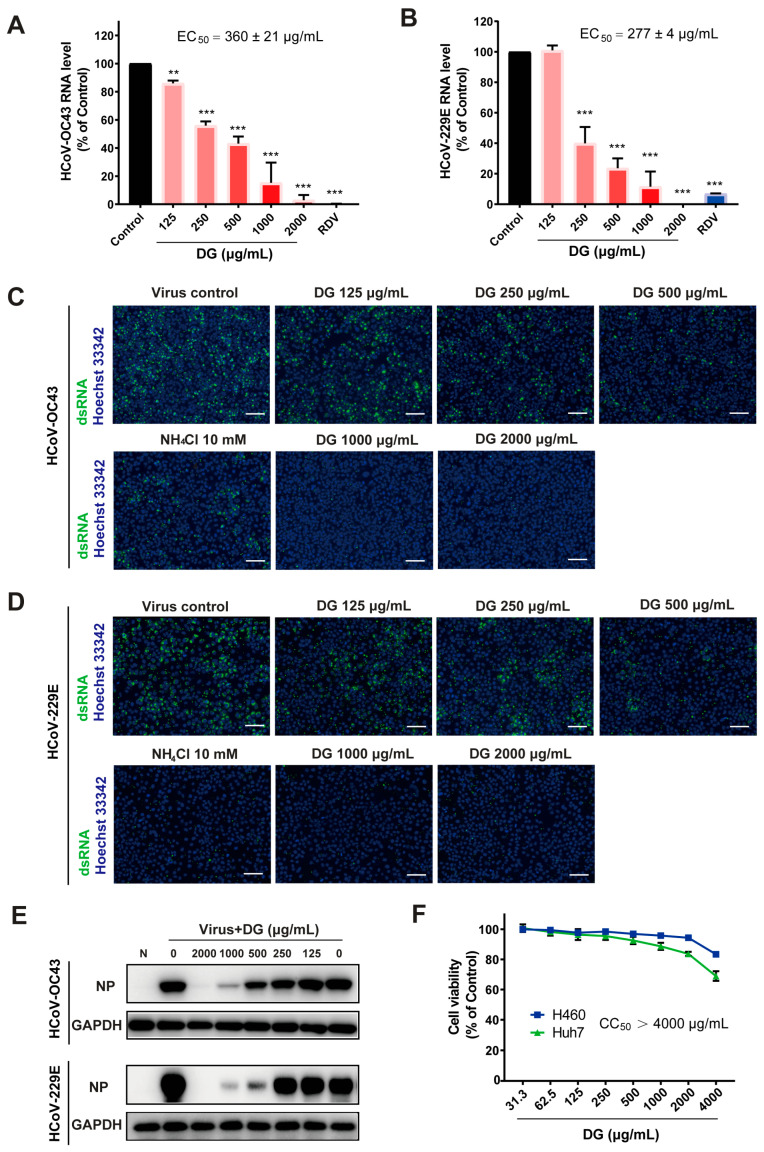
Diammonium glycyrrhizinate treatment significantly inhibited the replication of HCoV-OC43 and HCoV-229E. (**A**–**D**) H460 or Huh7 cells seeded in 24-well plates were infected with HCoV-OC43 or HCoV-229E (MOI = 0.05), and indicated concentrations of DG were added at the time of infection. RDV at 2 μM (**A**) or 0.05 μM (**B**) served as positive control. (**A**,**B**) At 24 h post treatment, the viral RNA levels were determined by a one-step qRT-PCR assay. Viral RNAs were normalized to GAPDH mRNA and presented as the percentage of infected control without treatment (Control). EC_50_ values were calculated by the Reed & Muench method. Values represented average results from two independent replicates (±SD). *p* values were calculated by one-way ANOVA. ** *p* < 0.01, *** *p* < 0.001 vs. control. (**C**,**D**) Amounts of double-stranded RNA in HCoV-infected cells were visualized by an immunofluorescent staining assay (scale bar: 200 μm). (**E**) Viral NP proteins were determined by a Western blot assay. (**F**) Cytotoxicity of DG in H460 and Huh7 cells was determined by a CellTiter-Fluo Cell Viability Assay.

To determine the role of DG in HCoV infection, the effects of DG on levels of HCoV-OC43 RNA in infected cells were first examined, with remdesivir (RDV) as positive control. The qRT-PCR results in Figure 1A,B indicate that incubation with DG in infected cells potently reduced viral RNA in HCoV-OC43 (EC_50_ = 360 ± 21 μg/mL) and HCoV-229E (EC_50_ = 277 ± 4 μg/mL) in a dose-dependent manner. The antiviral activity of DG was also confirmed by immunofluorescence staining and Western blot assays which detected viral dsRNA and nucleocapsid proteins, respectively, in infected cells (Figure 1C,E). NH_4_Cl, which can inhibit viral entry by elevating endosomal pH, also significantly inhibited infection with HCoV-OC43 or HCoV-229E (Figure 1C,D). Cytotoxicity of DG was determined in a CellTiter-Fluo Cell Viability assay, and CC_50_ values were greater than 4000 μg/mL (Figure 1F).

**Figure 2:**
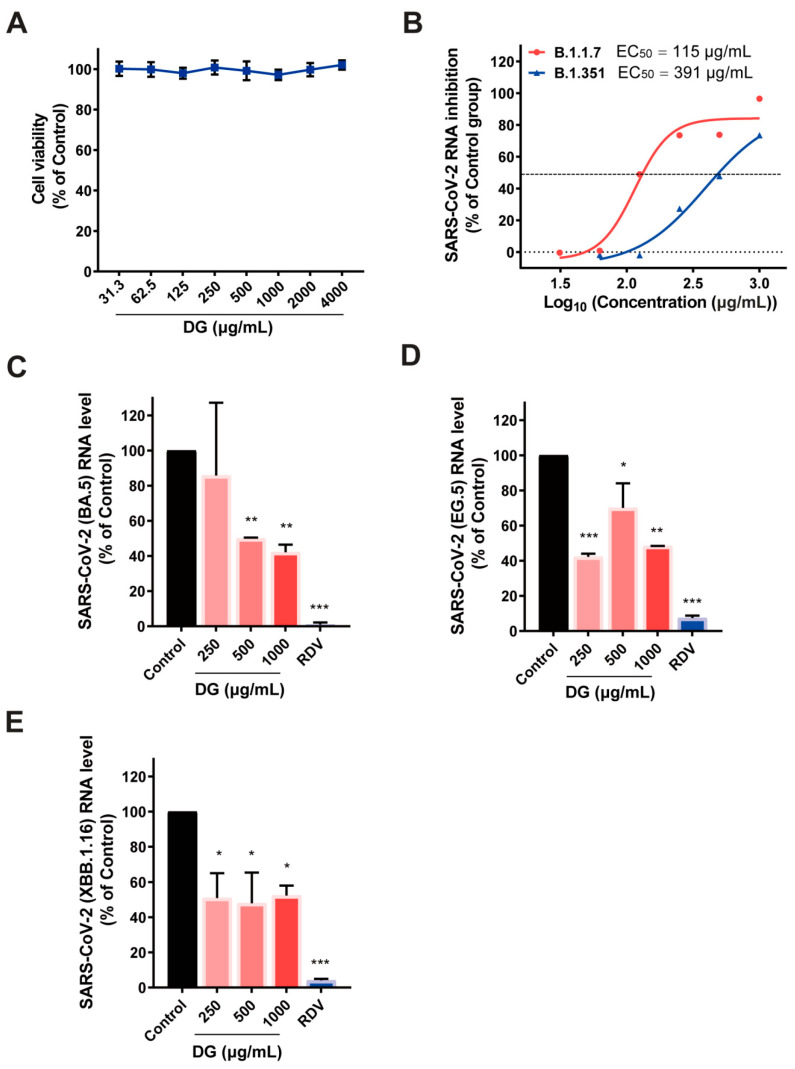
Diammonium glycyrrhizinate treatment significantly inhibited replication of SARS-CoV-2 variants B.1.1.7, B.1.351, BA.5, EG.5, and XBB.1.16. (**A**) The cytotoxic effect of DG on Vero E6 cells was determined by a CellTiter-Fluo Cell Viability Assay. (**B**–**E**) Vero E6 cells seeded in 96-well plates were pretreated with the indicated concentrations of DG for 1 h prior to infection with SARS-CoV-2 at an MOI of 0.05. At 24 h or 48 h post infection, total cellular RNA was extracted and the viral RNA levels were determined by a one-step qRT-PCR assay. Viral RNAs were normalized to GAPDH RNA and presented as a percentage of mock-treated control. Values represented average results of duplicated experiments. * *p* < 0.05, ** *p* < 0.01, *** *p* < 0.001 vs. control.

The antiviral effects of DG on SARS-CoV-2 were then assessed in Vero E6 cells. No cytotoxicity was observed in Vero E6 cells treated with DG up to 4000 μg/mL (Figure 2A). SARS-CoV-2 variants of concern (VOCs) are associated with increased transmissibility, elevated risk of reinfection, and/or reduced vaccine efficacy. Encouragingly, DG treatment significantly reduced Vero E6 infection with all the detected SARS-CoV-2 variants, including B.1.1.7, B.1.351, BA.5, EG.5, and XBB.1.16 (Figure 2B–E). These data indicate that DG exhibits potent antiviral activity against multiple coronaviruses.

### 2.2. Diammonium Glycyrrhizinate Exhibits Broad-Spectrum Interrupting Activity on Spike-Mediated Cellular Entry of HCoVs

**Figure 3:**
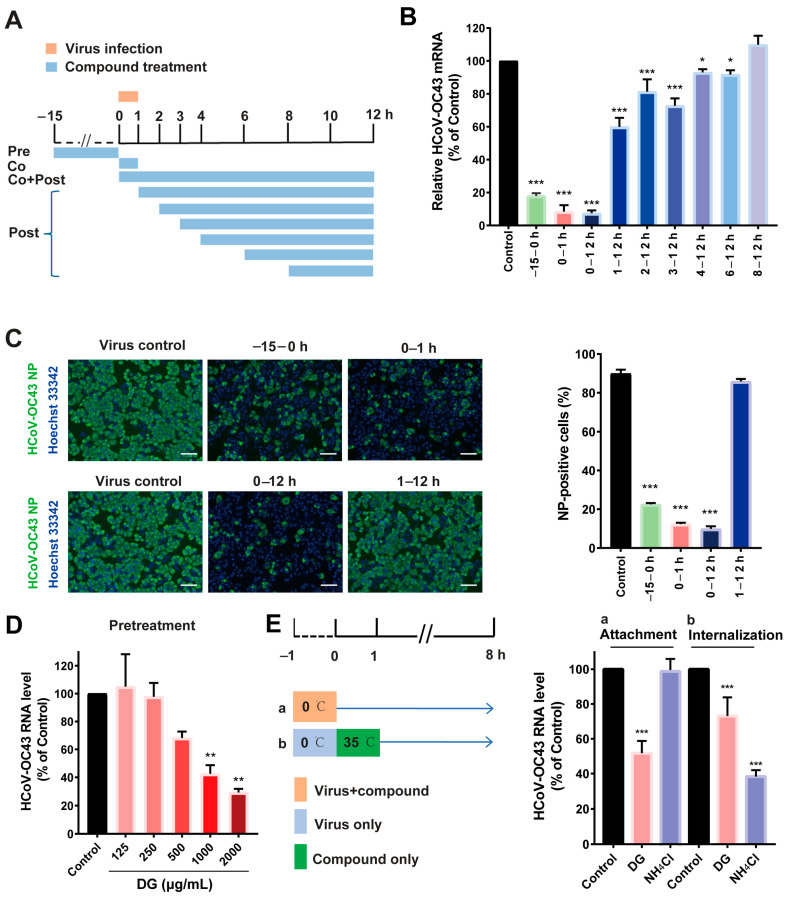
Diammonium glycyrrhizinate efficiently restricts HCoV-OC43 entry. (**A**–**C**) Time-of-addition analysis. H460 cells were infected with HCoV-OC43 at an MOI of 5 for 1 h. DG (2000 μg/mL) was present either in the 15 h before infection (−15 to 0) or during infection (0–1), or was added at the indicated time points until 12 h post-infection. (**B**) Total intracellular RNAs were extracted to determine the amounts of viral RNA in a qRT-PCR assay. Viral RNAs were normalized to GAPDH mRNA levels. The graph denotes the percentages of viral RNA relative to that of the mock-treated control added at the time of infection (time point 0). Each data point is a mean result of three independent replicates (± SD). *p* values were calculated by one-way ANOVA. * *p* < 0.05, ** *p* < 0.01, *** *p* < 0.001 vs. control. (**C**) Viral NP proteins were visualized by an immunofluorescent staining assay (scale bar: 200 μm). Percentages of NP-positive cells (green-positive cells/blue-positive cells) determined by randomly counting 3 images for the indicated time points are shown on the right. (**D**) H460 cells were pretreated with DG at indicated concentrations for 15 h. After removal of the compounds and two gentle washes, cells were infected with HCoV-OC43 at an MOI of 0.5. At 12 h post-infection, viral RNA was determined by a qRT-PCR assay. (**E**) Attachment: H460 cells were infected with HCoV-OC43 (MOI = 0.1) in the presence of DG (2000 μg/mL) or NH_4_Cl (10 mM) at 0 °C (on ice) for 1 h and then washed with PBS. Cells were then cultured with normal medium for 8 h. Internalization: H460 cells were infected with HCoV-OC43 (MOI = 0.1) at 0 °C (on ice) for 2 h and then washed with PBS. Cells were then cultured with medium containing 2000 μg/mL of DG or NH_4_Cl (10 mM) for 1 h at 35 °C. The infected cells were then treated with alkaline phosphate-buffered saline (PBS; pH 11) for 30 s to inactivate any viruses that had not penetrated the cells. Acidic PBS (pH 5) was then immediately added to neutralize the mix. The neutralized medium was then removed. Cells were then overlayed with normal medium and then incubated at 35 °C. Viral RNA was examined after 8 h of infection.

A time-of-addition experiment was performed during the HCoV-OC43 infection of H460 cells to assess the HCoV replication step (s) inhibited by DG (Figure 3A). As shown in Figure 3B, treatment of H460 cells with DG before infection (−15–0 h), during the virus inoculation period (0–1 h), and during full replication cycle (0–12 h), led to dramatic reductions in mRNA of 82%, 91.5%, and 92.6%, respectively, compared with the virus control group. A similar phenomenon was obtained in a parallel study that measured viral NP by immunofluorescence staining (Figure 3C). Addition of DG at 1 h post infection did not affect the number of nucleocapsid protein (NP)-positive cells (Figure 3C) and only moderately reduced the viral RNA levels (Figure 3B). These data indicate that DG predominately exerts its antiviral effect during the entry of HCoV infection. We further found that treatment of H460 cells with DG before infection could dose-dependently suppress viral RNA expression, confirming that pretreatment with DG significantly blocks HCoV infection (Figure 3D).

Next, to further explore the stage of viral entry at which DG works, the effects of DG on viral attachment and internalization were evaluated. As shown in Figure 3E, the amount of virus attached to the cells with DG treatment was significantly lower, compared to the control group. Nevertheless, the internalization assay showed that viral mRNA levels were slightly affected by DG treatment but dramatically altered by NH_4_Cl treatment. These results imply that DG probably blocks entry of HCoVs via a different mechanism to that of NH_4_Cl.

**Figure 4:**
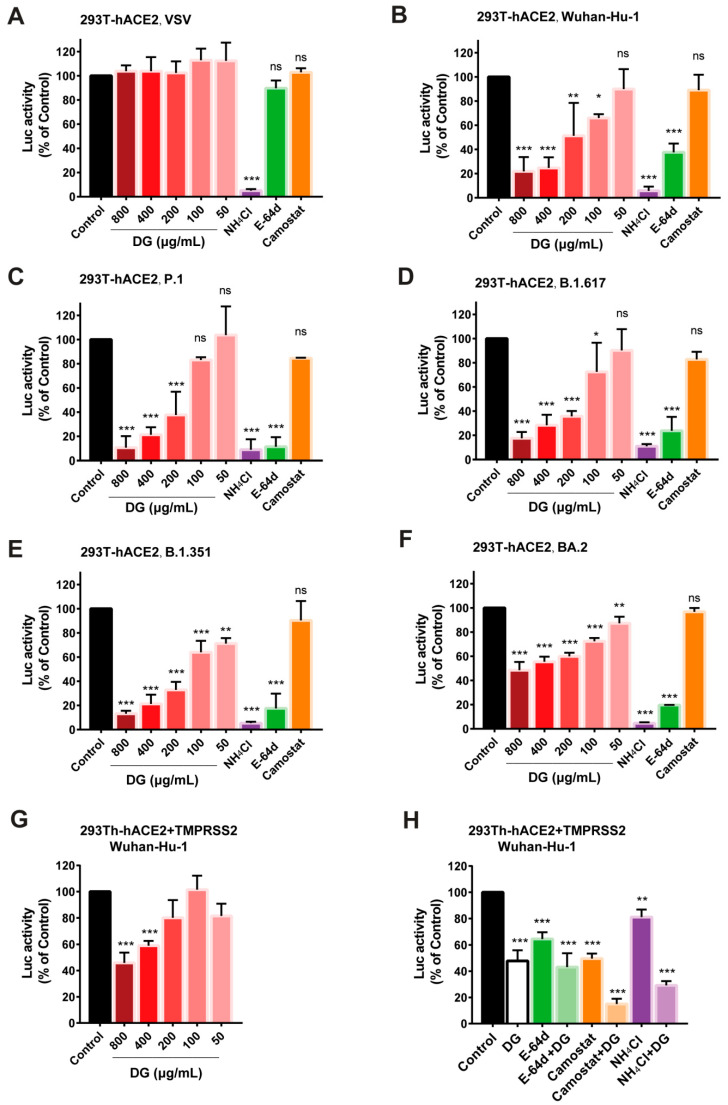
DG can dose-dependently inhibit the entry of SARS-CoV-2 pseudoviruses, including various variants of concern. (**A**–**F**) 293T-hACE2 cells seeded in 96-well plates were infected with VSV or indicated SARS-CoV-2 pseudoviruses in the presence of indicated concentrations of DG. As controls, 10 mM of NH_4_Cl, 1 μM of E-64d, and 50 μM of camostat were used. At 72 h post infection, the firefly luciferase activities were measured by microplate luminometry using a PerkinElmer EnSpire instrument. The luciferase activity was normalized to that of mock-treated control cells (*n* = 3). *, *p* < 0.05; **, *p* < 0.01; ***, *p* < 0.001. ns—not significant. (**G**,**H**) TMPRSS2 overexpression can partially rescue the inhibitory effect of DG on infection by SARS-CoV-2 pseudoviruses (**G**); and combination of DG with other coronavirus inhibitors enhances their antiviral activities (**H**). 293T-hACE2 cells transiently expressing TMPRSS2 were infected with SARS-CoV-2 pseudovirus in the presence of DG (50–800 μg/mL) (**G**) or in the presence of DG (800 μg/mL), E-64d (50 μM), E-64d (50 μM) + DG (800 μg/mL), camostat (50 μM), camostat (50 μM) + DG (800 μg/mL), NH_4_Cl (10 mM), or NH_4_Cl (10 mM) + DG (800 μg/mL) (**H**). At 72 h post infection, the firefly luciferase activities were measured by microplate luminometry using a PerkinElmer EnSpire instrument. The luciferase activity was normalized to that of mock-treated control cells (*n* = 3).

We then sought to determine whether DG could also inhibit the cellular entry of SARS-CoV-2 by using a pseudotyped lentivirus system. We found that DG treatment at non-toxic doses could dose-dependently reduce infection with pseudotyped SARS-CoV-2, as well as various variants of concern (Wuhan-Hu-1, P.1, B.1.617., B.1.351, and BA.2), but had no effect on VSV pseudovirus in hACE2-expressing 293T cells (Figure 4A–F and Figure S1), indicating that DG could specifically block viral S protein-mediated entry by ACE2. In agreement with antiviral mechanisms and with previous observations [19], we found the following: NH_4_Cl, which can elevate endosomal pH, prevented infection with either pseudovirus; E-64d, which targets cathepsin B/L, was only active against spike protein-pseudotyped virus; and camostat, an inhibitor of TMPRSS2, did not alter the entry of either pseudovirus in 293T-hACE2 cells in which TMPRSS2 protein was marginally expressed.

TMPRSS2 is a protease reported to participate in the proteolytic processing of SARS-CoV-2 S protein [19]. Activation of the S protein by TMPRSS2 in close proximity to the ACE2 receptor initiates fusion between the viral membrane and the plasma membrane. We analyzed whether overexpression of TMPRSS2 could reverse the inhibition of DG on SARS-CoV-2-S-driven entry into cells. 293T-hACE2293T cells transiently expressing TMPRSS2 were infected with pseudotyped SARS-CoV-2 in the presence of DG. As shown in Figure 4G, the inhibition of DG on luciferase activity in TMPRSS2^+^ 293T-hACE2 cells was less efficient than in 293T-hACE2 cells (Figure 4B). Similarly, overexpression of TMPRSS2 also rescued SARS-CoV-2-S-driven entry from inhibition by E-64d or NH_4_Cl (Figure 4H), consistent with a previous report [19]. Moreover, camostat treatment significantly blocked SARS-CoV-2-S-driven entry into the TMPRSS2^+^ 293T-hACE2 cells (Figure 4H). Interestingly, much stronger inhibition of SARS-CoV-2 pseudotyped virus activity was attained when DG was combined with camostat and NH_4_Cl (Figure 4H), indicating that a combination of DG with cathepsin B/L or TMPRSS2 inhibitor might exhibit synergy in the inhibition of SARS-CoV-2 S-driven cell entry.

Overall, then, the results indicated that DG efficiently inhibited infection by any of multiple coronaviruses by targeting spike protein-mediated entry events.

### 2.3. Diammonium Glycyrrhizinate Specifically Binds to the RBD Fragment to Block Interaction Between the SARS-CoV-2 Spike and Human ACE2

**Figure 5:**
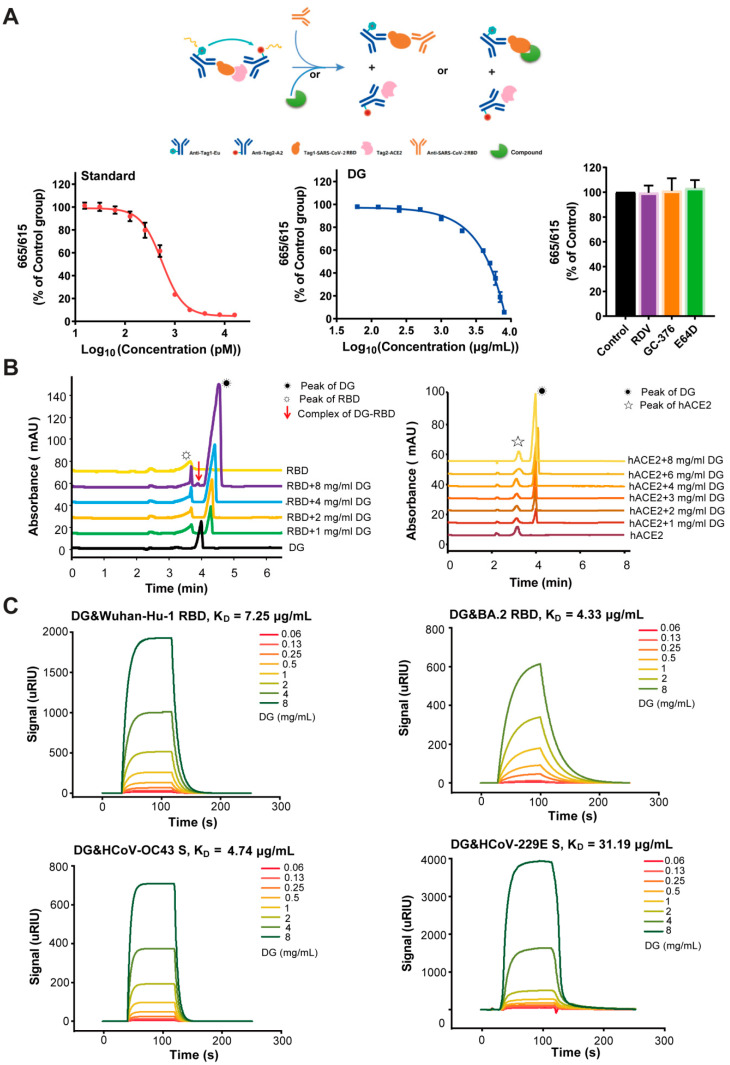
Diammonium glycyrrhizinate efficiently blocks interaction between SARS-CoV-2 spike protein and human ACE2 by binding to the RBD. (**A**) The interaction of SARS-CoV-2 S protein RBD and hACE2 was detected by a TR-FRET-based interaction assay. (**B**) CE spectrogram of RBD-DG interaction (**left panel**) and hACE2-DG interaction (**right panel**). Concentration of RBD was maintained at 2 μM while concentration of DG was increased in the interaction system. The DG peak marked with a shining sun appears at 4:10 min, and the RBD peak marked with a star appears at 3:75 min. Concentration of hACE2 was maintained at 1 μM while concentration of DG was increased in the interaction system. The DG peak marked with a shining sun appears at 4:00 min, and the hACE2 peak marked with a star appears at 3:15 min. (**C**) SPR analysis of the interaction between DG and RBD of SARS-CoV-2 (Wuhan-Hu-1 and BA.2) or S protein of HCoV-OC43 and HCoV-229E.

The spike protein of HCoVs plays an essential role in viral attachment, fusion, entry, and transmission. The RBD in the N-terminal S1 subunit is responsible for virus–receptor binding [20]. As DG mainly reduced the attachment of HCoV, we then investigated whether DG could block the interaction between the RBD fragment of SARS-CoV-2 and human ACE2, thereby interrupting viral entry, by using a TR-FRET-based SARS-CoV-2 S-RBD-ACE2 interaction assay. The neutralizing antibody targeting the RBD of SARS-CoV-2 S protein was included as a standard control. The schematic diagram of TR-FRET is depicted in Figure 5A. Notably, DG efficiently impaired the interaction between the RBD and human ACE2 proteins (Figure 5A) in a concentration-dependent manner. Contrarily, other non-targeting S protein compounds, including RDV, GC-376, and E-64d, had no effect on the binding of RBD to human ACE2 (Figure 5A).

In order to further identify the mechanism of DG-mediated inhibition of the RBD-ACE2 interaction, we next analyzed interactions between DG and RBD and between DG and hACE2. Recombinant RBD or hACE2 protein was incubated with DG at RT for 15 min, and then injected into CE for analysis. Strikingly, the RBD peak height increased with increasing additions of DG to the interaction system (Figure 5B, left panels). In particular, when DG reached 8 mg/mL, the peak RBD-DG complex appeared at 3.90 min, indicating that the binding between DG and RBD was so strong that a stable complex was formed (Figure 5B, left panels). However, no obvious interaction was observed in CE analysis of the interaction of DG with hACE2 (Figure 5B, right panels), indicating that DG can directly bind to the RBD but not to hACE2. The binding activities of DG to the SARS-CoV-2 RBD were also detected using a SPR technique. The interactions between DG and RBD were concentration-dependent, and DG showed high affinity for the RBDs of SARS-CoV-2 strains Wuhan-Hu-1 and BA.2, with equilibrium dissociation constants (*K_D_*) of 7.25 and 4.33 μg/mL, respectively, being recorded (Figure 5C). Taken together, these results indicate that DG specifically targets RBD and thus effectively blocks the interaction between the SARS-CoV-2 spike and human ACE2.

We then investigated the binding activity of DG to the S proteins of HCoV-OC43 and HCoV-229E through SPR assays. As shown in Figure 5C, DG obviously reacted with both S proteins, with *K_D_* values of 4.74 and 31.19 μg/mL, respectively, being recorded. Taken together, these results suggest that DG exhibits broad-spectrum binding activity to the spike protein of human coronaviruses to block interactions between spikes and their respective receptors, thereby interrupting viral entry.

### 2.4. Molecular Docking Analysis Determines the Binding Sites of DG and HCoV RBD Complexes

**Figure 6:**
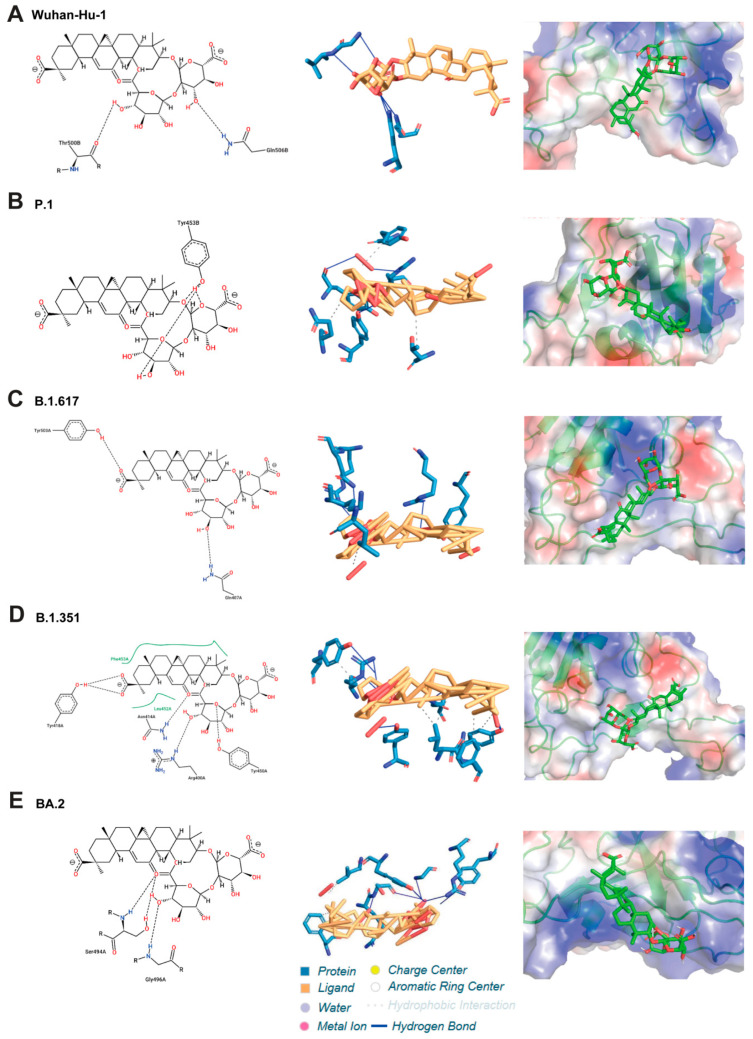
Two-dimensional structure of diammonium glycyrrhizinate and three-dimensional illustration of its interaction with the hACE2 binding pocket of RBD of SARS-CoV-2. (**Left panels**) Representation of 2D structure of DG in complex. The blue and green spheres represent residues involved in hydrogen bond interactions and the salt bridge, respectively. (**Middle panels**) Hydrogen bonds in the protein–ligand complex are shown as yellow dotted lines. (**Right panels**) 3D illustration of DG (stick representation) in the binding pocket of SARS-CoV-2-RBD (surface representation) (PDB ID: 6vw1).

To further characterize the binding effect and determine the sites of DG and HCoV RBD complexes, we then performed computer simulation and molecular docking. The RBDs of five SARS-CoV-2 strains (Wuhan-Hu-1, B.1.351, B.1.617, P.1, and BA.2), HCoV-229E, and HCoV-OC43 were simulated to analyze their binding regions and the possible interaction mechanisms. As shown in Figure 6 and Figure S2, the 3D model of DG and RBD complexes was simulated and refined by molecular dynamics. It was observed that DG mainly binds to the receptor binding pocket of RBDs, suggesting that DG has the potential to block RBD recognition and bind its receptor. The interacting residues and the types of interaction force in the RBD/DG complexes are shown in Tables S1–S7. The EC_50_ of DG showed a similar order of magnitude in the virus infection inhibition assay, whereas the stability of DG complex with HCoV-OC43 or HCoV-229E was higher than that of SARA-CoV-2 in the simulation results. DG binds to the amino acid residues of RBDs mainly by hydrogen bonding and hydrophobic interaction, and the number of interacting residues varies from 6 to 11.

In addition, cross-interacting amino acid residues in the SARS-CoV-2 RBD/ACE2 complexes, RBD/DG complexes, and mutations on RBD of VOCs were analyzed (Figure S3). Because the key amino acid residues in the binding interface between SARA-CoV-2 RBD and ACE2 were identified as Tyr449, Tyr453, Lus455, Phe456, Ala475, Gly476, Phe486, Asn487, Tyr489, Gln493, Gly496, Gln498, Thr500, Asn501, Gly502, and Tyr505 [21], we identified that DG mainly interacts with Y449, G502, Y453, Q493, and Y505 at the RBD/ACE2 binding interface. Consistently, purified RBD containing four mutated amino acids (Y449A, Y453A, Q493A, and G502A) showed a great decrease in binding activity to DG, with a *K_D_* value of 18.08 mg/mL being recorded (Figure S4). For the SARS-CoV-2 variants B.1.351, B.1.617.2, and P.1 [22,23,24], DG did not bind to the mutation sites, but to the conserved sites, possibly providing DG with broad-spectrum resistance to SARS-CoV-2. However, in the case of Omicron BA.2, with 15 mutations in its RBD [24], in addition to maintaining interaction with the conserved residue Y679, DG combined with four additional mutant residues, N501Y, G496S, Q498R, and Q493R, to generate a hydrogen bond network. Overall, our studies showed that DG may directly bind to a conserved hydrophobic pocket of the RBD in the spike protein of human coronaviruses.

### 2.5. Intranasal Administration of Diammonium Glycyrrhizinate Significantly Protects Mice from HCoV-OC43 Infection

**Figure 7:**
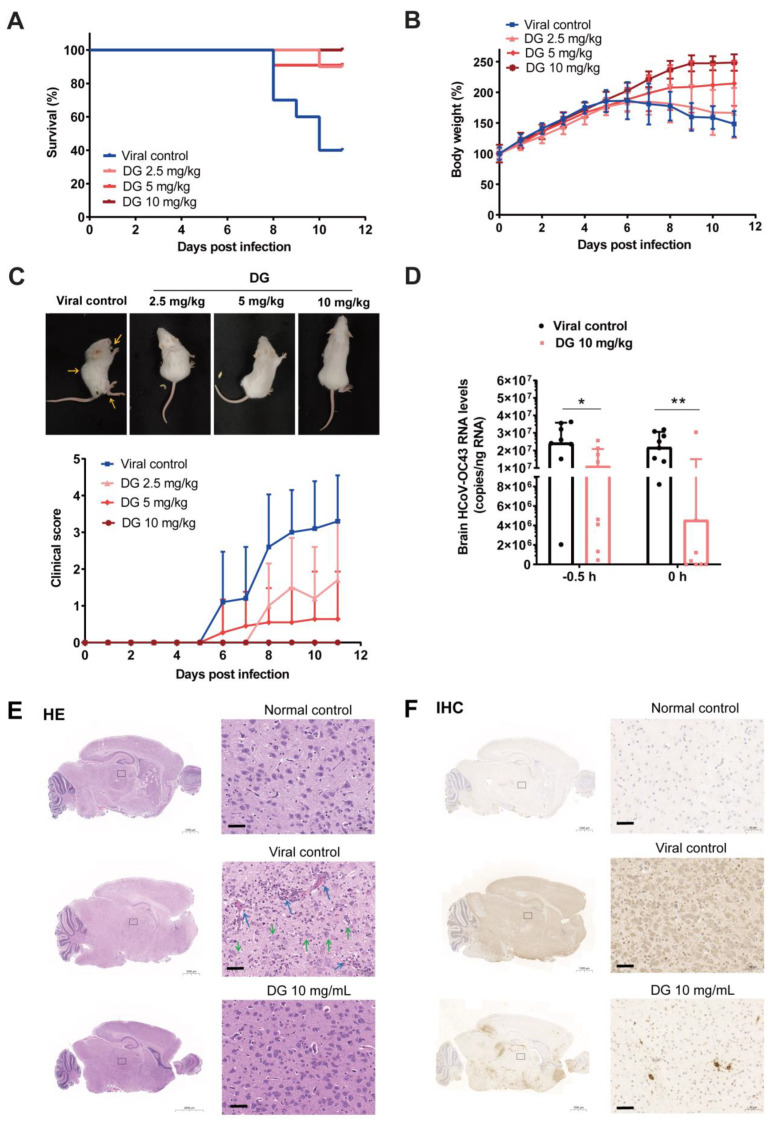
Intranasal administration of DG effectively inhibited HCoV-OC43 infection in mice. (**A**–**C**) Suckling Balb/c mice aged 4–6 days were administered intranasally with DG (2.5 mg/kg, 5 mg/kg or 10 mg/kg) in sterile deionized water simultaneously with the challenge of HCoV-OC43 (1000 TCID_50_). For mice in the viral control group, the same volume of sterile deionized water was administered intranasally. Each group contained ten mice. Mice survival (**A**), body weight change (**B**), and clinical scores (**C**) were monitored every day until day 11. Images of representative mice were taken at 11 dpi (**C**, yellow arrow, paralysis of forelimbs and hindlimbs with an arched back). (**D**–**F**) Suckling Balb/c mice aged 6 days were intranasally administered DG (10 mg/kg) in sterile deionized water 0.5 h before (−0.5 h) or simultaneously with (0 h) the challenge of HCoV-OC43 (1000 TCID_50_). Each group contained eight mice. All the mice were euthanized on day 6 after infection. Viral RNA levels in mouse brain were then determined by a RT-qPCR assay (**D**). Hematoxylin and eosin (H&E) staining of brain tissues from viral control or DG-treated HCoV-OC43 infected mice (0 h) was conducted (**E**). Blue arrows represent vascular congestion, or visible proliferation and infiltration of glial cells around the blood vessels; green arrows represent visible neuronal atrophy and reduction, increased cell degeneration, or neurophilia. Levels of viral N protein in brain tissues (0 h) were determined by immunohistochemistry (**F**). Representative images are presented to highlight the predominant staining patterns found in each mouse. Scale bar = 50 μm. *, *p* < 0.05; **, *p* < 0.01.

Considering that DG mainly inhibits entry of HCoVs, we then used a HCoV-OC43 infection mouse model to further investigate the potential protective effect of DG through intranasal administration. Firstly, we treated suckling mice with DG (2.5 mg/kg, 5 mg/kg, or 10 mg/kg) intranasally in sterile deionized water simultaneously with the challenge of HCoV-OC43 at 1000 TCID_50_. In the viral control group, mice started to die on day 8 after infection. The final survival rates of mice in the viral control, DG-2.5 mg/kg, DG-5 mg/kg, and DG-10 mg/kg groups were 60%, 90%, 90%, and 100%, respectively (Figure 7A), indicating that intranasal administration of DG significantly reduced the lethality from the HCoV-OC43 challenge. Moreover, compared with the viral control group, DG treatment also significantly alleviated weight loss in infected mice (Figure 7B), and led to lower clinical manifestation scores (Figure 7C) from HCoV-OC43 infection. As shown in Figure 7C, DG significantly attenuated HCoV-OC43-induced upper- and hind-limb paralysis, as well as ataxia, on day 11 after infection. These results indicate that intranasal administration of DG significantly reduced HCoV-OC43 infection in mice.

Because HCoV-OC43 infection can damage the central nervous system (CNS) in mice, leading to neurological disease and consequent disabilities, we next examined the effect of DG on viral propagation and histopathology in brains of HCoV-OC43-infected mice. After 6 days of infection, viral RNA levels in the brains of mice subjected to DG treatment with a single dose of 10 mg/kg via nasal administration either 0.5 h before (−0.5 h) or simultaneously with (0 h) with HCoV-OC43 infection significantly decreased, similarly to the viral control group (Figure 7D). HE staining analysis indicated that brain tissues in the DG group (0 h) exhibited apparent histopathological changes, including vascular congestion, inflammation infiltration, neuronal atrophy and reduction, and vacuolation and degeneration (Figure 7E). IHC studies demonstrated that HCoV-OC43 N protein expression in virus-infected brains was significantly reduced in DG-treated mice (0 h) compared with viral control mice (Figure 7F). Taken together, these results thus indicated that diammonium glycyrrhizinate has a significant protective effect against HCoV infection in vivo.

### 2.6. Combinations of Diammonium Glycyrrhizinate and Other HCoV Inhibitors Produce Synergistic Antiviral Effects

**Figure 8:**
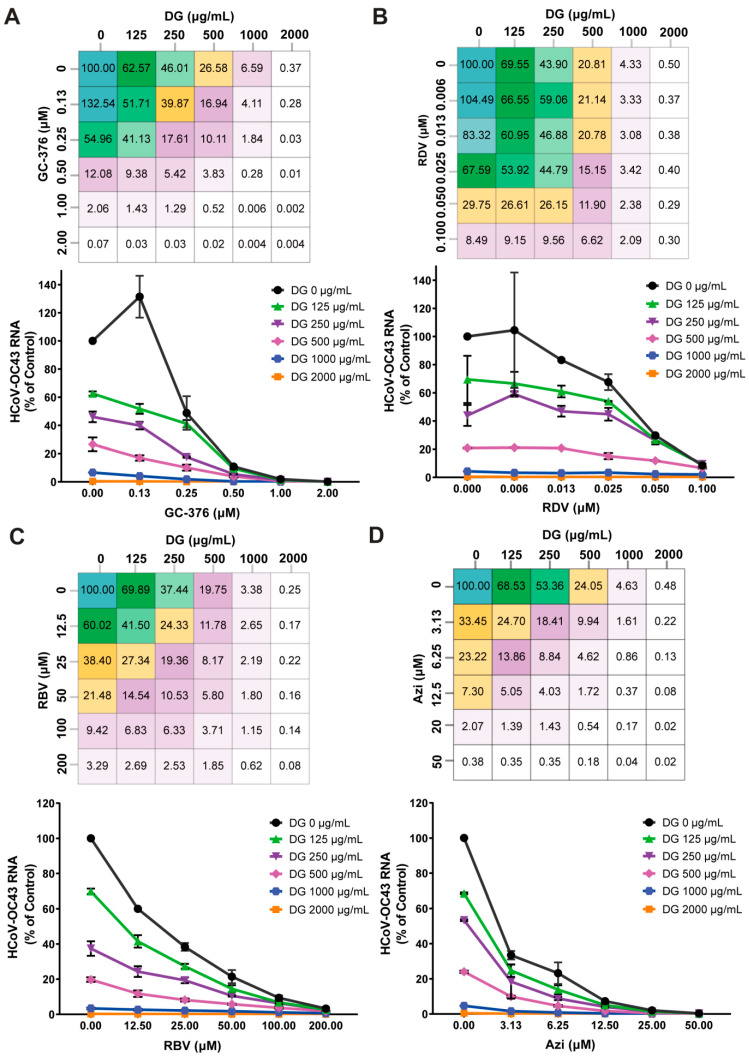
Combination of DG with other coronavirus inhibitors enhances antiviral activities. H460 cells seeded in 24-well plates were infected with HCoV-OC43 (MOI = 0.05) and indicated concentrations of GC-376 (**A**), RDV (**B**), RBV (**C**) and azithromycin (Azi) (**D**), either alone or in combination with DG, were added at the time of infection. At 24 h post-treatment, the viral RNA levels were determined by a one-step qRT-PCR assay. Viral RNAs were normalized to GAPDH mRNA and presented as the percentage of mock-treated control. Dose matrix-response values for the combination, showing relative HCoV-OC43 RNA levels for the serially diluted compounds.

Drug cocktails or combinations have been utilized more commonly in recently reported antiviral therapies [25,26]. The effects of combining DG with other coronavirus inhibitors, including GC-376, RDV, RBV, and azithromycin, were tested in HCoV-OC43-infected H460 cells. Each of these inhibitors has a distinct mode of action. To assess whether synergy could be achieved, H460 cells were infected with HCoV-OC43 in the presence of various concentrations of selected compounds, either alone or in combination with DG, and antiviral activities were determined by measuring viral mRNA levels. As shown in Figure 8, dose-dependent inhibition was observed for all of them when used alone or in in any combinations of two. The data also showed that the DG-GC-376, DG-RBV, and DG-azithromycin pairs exhibited strong synergy, but there was no obvious synergistic antiviral effect of DG with RDV. Collectively, these results demonstrate that combinatory treatments of DG and distinct classes of HCoV inhibitors have synergistic or additive antiviral effects.

## 3. Discussion

Despite the recent approval of several drugs to treat COVID-19, there is still a great medical need for agents with a broad-spectrum anti-coronavirus effect to prevent the spread of any emerging epidemic. Glycyrrhizic acid and its derivatives have been reported to have potent pharmacological effects against viral infections including SARS-CoV and SARS-CoV-2. However, their antiviral mechanisms against coronavirus are not fully understood.

In this study, we found that diammonium glycyrrhizinate exists potent antiviral activities against HCoV-229E and HCoV-OC43, and SARS-CoV-2 (Figure 1 and Figure 2). It seems that DG exhibits similar anti-coronavirus activity to glycyrrhizin in vitro [27,28]. Given that DG has also been found to be active against animal coronaviruses such as infectious bronchitis virus (IBV) [29] and porcine epidemic diarrhea virus (PEDV) [30], it is possible that DG has the potential to serve as a broad-spectrum inhibitor against both human and animal coronavirus.

A time-of-addition assay of HCoV-OC43-infected H460 cells and pseudotyped lentiviral infection studies demonstrated that DG potently inhibited infection by any of multiple HCoVs by targeting one or multiple entry replication events (Figure 3 and Figure 4). Coronavirus entry into cells occurs in three steps: (1) binding of virus to the host cells, (2) activation of spike protein by proteolysis, and (3) spike-mediated fusion. Currently, many studies are focusing on the development of small molecules or peptides/antibodies that target the spike protein or its receptor hACE2 [31]. Our viral attachment and internalization assays revealed that DG predominantly inhibits virus attachment to cells, which is consistent with the results of a recent report showing that DG treatment led to a significant reduction in PEDV mRNA levels in viral attachment assays [17,30].

Molecular docking has predicted that DG inhibits the interaction of RBD with ACE2 [32]. In this study, we obtained experimental evidence that DG effectively prevents binding of the RBD domain of SARS-CoV-2 spike protein to the hACE2 receptor (Figure 5 and Figure S4). Glycyrrhizic acid also exerts binding activity with the S1 subunit of MERS-CoV and SARS-CoV-2 [16,33]. It is likely that glycyrrhiza species can utilize the binding of S protein against SARS-CoV-2 infection and disease. However, another docking result showed that glycyrrhizin has the potential to bind to ACE2 [34]. Through CE or SPR assays, we demonstrated that DG had no binding activity to human ACE2 but specifically interacted with the RBD of SARS-CoV-2 with high affinity, thereby blocking their interaction. In addition, we also demonstrated that DG interacts with the spike protein of HCoV-OC43 or 229E, indicating that DG exhibits a broad-spectrum binding activity to the spike protein of human coronaviruses to block the viral entry.

Binding to highly conserved epitopes of RBD can potently neutralize many currently circulating SARS-CoV-2 variants [6]. The potential binding pockets of DG on RBD of various coronavirus S protein, as determined by our computational molecular docking and amino acid mutation analysis, demonstrated that DG mainly interacts with Y449, G502, Y453, Q493, and Y505 at the RBD/ACE2 binding interface (Figure 6, Figures S2–S4). DG retained its antiviral activity against the several SARS-CoV-2 variants (Figure 2 and Figure 4), indicating that most of the predicted contact residues in the DG-binding region may be conserved.

DG also possesses anti-inflammatory and antioxidant effects [35,36]. Because the DG was administrated simultaneously with HCoV-OC43 infection in this study, the inhibitory effect of DG on brain inflammation in suckling mice should have been due to its inhibition of viral entry rather than any direct inhibition of inflammation (Figure 7). In the future, it is necessary to further research the inhibitory effect of DG on inflammation induced by coronavirus infection in vivo, by such means as changing the administration times or frequency.

A randomized, open, controlled trial (www.chictr.org.cn/, China Clinical Trial Registry number: ChiCTR2000029768 and ChiCTR2000030490) showed that diammonium glycyrrhizinate enteric-coated capsules (DGECs) were not associated with statistically significant clinical benefits in COVID-19 patients. However, some reports have shown that a combination of DG and VC can relieve severe symptoms from COVID-19 [37,38]. In addition, another report identified that abnormal liver enzyme activities were related to SARS-CoV-2-induced acute liver damage, and that DG treatment might alleviate abnormal liver functions in non-critical COVID-19 patients [39]. Thus, the question of whether DG can relieve severe symptoms and reduce mortality in COVID-19 cases needs to be addressed in studies with a larger cohorts.

Drug delivery by inhalation is an important strategy in treating lung diseases. The respiratory tract is the primary target tissue of HCoV infection; thus, the nasal route seems a preferred choice for drugs that inhibit viral invasion. It is worth studying and developing new dosage form for aerosol delivery of DG in the future. Because coronavirus primarily spreads through respiratory droplets containing the virus, our results, which indicate that DG exhibits a broad-spectrum binding ability to the spike protein of coronavirus and intranasal application of diammonium glycyrrhizinate significantly protected of mice from HCoV-OC43 infection, encourage study of the efficacy of nebulized DG inhalation for the treatment of coronavirus in the future.

Although the combination antiviral approach needs to be improved, the encouraging in vitro synergistic antiviral effects of DG in combination with other inhibitors (Figure 8) suggest that a combination of these might be a good candidate for alternative medicine against COVID-19. Future studies are needed to expand on the findings of the present work in in vivo studies.

## 4. Materials and Methods

### 4.1. Cells and Viruses

Huh7 (Human hepatocellular carcinoma cell lines) and H460 (human lung cancer cell line) were kindly provided by Dr. Zonggen Peng and Dr. Zhen Wang, respectively, at the Institute of Medicinal Biotechnology, Chinese Academy of Medical Sciences and Peking Union Medical College. C3A (Human hepatoblastoma cell line) was purchased from ATCC. 293T-derived cell line expressing human ACE2 (293T-hACE2) was purchased from Delivectory Biosciences Inc. (Beijing, China). African green monkey kidney cell line Vero E6 was kindly provided by the Institute of Medical Biology at the Chinese Academy of Medical Science (Kunming, China). All cells were cultured in Dulbecco’s Modified Eagle Medium (DMEM, Invitrogen, Carlsbad, CA, USA) or Minimum Essential Media (MEM, Invitrogen, Carlsbad, CA, USA) supplemented with 10% FBS and antibiotics (100 U/mL penicillin and 100 mg/mL streptomycin) at 37 °C.

HCoV-229E (VR740) was purchased from ATCC. HCoV-OC43 (VR1558) was provided by Dr. Xuesen Zhao at Beijing Ditan Hospital, Capital Medical University. SARS-CoV-2 B.1.1.7, B.1.351, BA.5 EG.5, and XBB.1.16 variants were stored at the P3 laboratory of the National Kunming High-Level Biosafety Primate Research Center, Institute of Medical Biology, Chinese Academy of Medical Sciences and Peking Union Medical College, Yunnan, China. Lentiviruses pseudotyped with VSV glycoprotein (VSV-G) or SARS-CoV-2 spike protein were obtained from Delivectory Biosciences Inc. (Beijing, China) and Vazyme Biotech Co., Ltd. (Nanjing, China).

### 4.2. Compounds and Plasmids

Diammonium glycyrrhizinate was purchased from TargetMol (Boston, MA, USA) and Selleck Chemicals (Houston, TX, USA). Remdesivir (RDV), Ribavirin (RBV), azithromycin, and camostat were purchased from TargetMol (Boston, MA, USA). E-64d and ammonium chloride (NH_4_Cl) were purchased from MedChemExpress (Monmouth Junction, NJ, USA).

The human transmembrane serine protease 2 (TMPRSS2) sequence was synthesized via GenScript and cloned into the pcDNA3.1(+) plasmid to generate the plasmid pcDNA3.1(+)-TMPRSS2.

### 4.3. Cell Cytotoxicity Assay

The cytotoxicity of compounds on different cells was determined by the CellTiter-Fluo Cell Viability Assay (Promega). Briefly, cells seeded into 96-well plates (2 × 10^4^/well) were treated with DG at different concentrations (starting from 4000 μg/mL and diluted by half each time) in triplicate. At 24 or 48 h post treatment, CellTiter-Fluo Reagent was added and wells were incubated at 37 °C for 30 min. The fluorescence was measured by a fluorometer (380 nm excitation/505 nm emission) with an EnSpire instrument (Perkin Elmer, Waltham, MA, USA). Concentration of cytotoxicity 50% (CC_50_) was calculated using GraphPad Prism Software (Versions 7 and 9).

### 4.4. Antiviral Activity Assay

H460 cells and Huh7 cells seeded in 24-well plates were infected with HCoV-OC43 or HCoV-229E (MOI of 0.05), respectively, and the indicated concentrations of compounds were added simultaneously. Antiviral efficacy was evaluated by analysis of viral RNA or protein levels at 24 h post infection.

For inhibition of authentic SARS-CoV-2 infection, Vero E6 cells seeded in 96-well plates were pretreated with different concentrations of DG for 1 h and infected with SARS-CoV-2 variants at an MOI of 0.05. Antiviral efficacy was evaluated by analysis of viral RNA levels at 24 h or 48 h post infection. EC_50_ values were calculated using GraphPad Prism Software.

### 4.5. RNA Extraction and qRT-PCR Assay

Total cellular RNA was extracted using TRIzol (Invitrogen) and FineMag rapid magnetic-bead virus DNA/RNA extraction reagents using the automated extraction instrument Purifier 32 (Genfine Biotech, Beijing, China) according to the manufacturer’s instructions. Amounts of viral RNA were determined using a quantitative reverse-transcription polymerase chain reaction (qRT-PCR) assay with TransScript Probe One-Step qRT-PCR SuperMix (TransGen, Beijing, China) (for HCoV-OC43 and SARS-CoV-2 detection) and HiScript II One Step qRT-PCR SYBR Green Kit (Vazyme, Nanjing, China) (for HCoV-229E detection) with the ABI 7500 Fast Real-Time PCR system (Applied Biosystems, Foster City, CA, USA). GAPDH was also quantified to normalize the levels of viral RNA. The detailed primers used in this study have been described previously [40].

### 4.6. Immunofluorescence Assay

Cells fixed with 4% paraformaldehyde were permeabilized in 0.5% Triton X-100 for 15 min and blocked in PBS containing 1% BSA for 60 min at room temperature (RT). Cells were then incubated overnight at 4 °C, either with an antibody against HCoV-OC43 nucleocapsid protein (Millipore) or with a dsRNA antibody (SCICONS, Szirák, Hungary), at a dilution of 1:200. After routine washing, the samples were incubated with Alexa Fluor 488-labeled goat anti-mouse secondary antibody (Beyotime, Shanghai, China) at room temperature for 1 h. The nucleus was stained with Hoechst 33342 (Beyotime, Shanghai, China). After washing with PBS, images were taken by a fluorescence microscope (Axio Observer, ZEISS, Oberkochen, Germany).

### 4.7. Western Blot Assays

Cellular proteins were extracted from cell lysates using M-PER Mammalian Protein Extraction Reagent (Thermo Fisher Scientific, New York, NY, USA) containing halt protease inhibitor single-use cocktail and then denatured in protein loading buffer at 100 °C for 10 min. Immunoblotting for HCoV-OC43 nucleocapsid protein (Millipore, Billerica, MA, USA, 1:1000) and HCoV-229E nucleocapsid protein (Sino Biological, Beijing, China, 1:1000) was performed as described previously [40]. GAPDH (Cell Signaling Technology, Danvers, MA, USA 1:1000) was used as a loading control.

### 4.8. Time-of-Addition Assay

To illustrate the effect of DG on the steps of the virus replication cycle, a time-of-addition experiment was performed. Briefly, H460 cells were infected with HCoV-OC43 at a multiplicity of infection (MOI) of 5 at 35 °C. DG was added 15 h before (pre-treatment), simultaneously with (co-treatment), or at different time points after (post-treatment) virus inoculation. At 12 h post-infection, cells were harvested and viral mRNA levels were determined by a one-step qRT-PCR assay. Viral nucleocapsid protein (NP) was also detected by the immunofluorescence assay.

### 4.9. Attachment and Internalization Assays

To determine the specific stage of the viral entry process influenced by DG, attachment and internalization assays were performed. Briefly, for the attachment assay, precooled H460 cells were infected with HCoV-OC43 in the presence or absence of DG (2000 µg/mL) on ice for 1 h. The medium was then removed and the cells washed three times with precooled PBS before fresh medium was added. After 8 h of incubation at 35 °C, the viral mRNA levels were detected by qRT-PCR.

For the internalization assay, precooled H460 cells were infected with HCoV-OC43 on ice for 1 h. After the medium containing the unbound virus was removed, cells were washed three times with precooled PBS and then incubated with DG (2000 µg/mL) at 35 °C for 1 h to trigger endocytosis of the virus. The infected cells were then treated with alkaline PBS (pH = 11) for 30 s to inactivate any viruses that had not penetrated the cells, then with acidic PBS (pH = 3) to neutralize the mix. The cells were then washed and cultured in fresh medium for another 7 h at 35 °C for viral mRNA detection.

### 4.10. Pseudovirus Infection and Luciferase Assay

A pseudovirus infection and luciferase assay was performed as previously described [40]. Briefly, 293T-hACE2 cells seeded into white-wall, clear-bottom 96-well plates were infected with lentiviruses pseudotyped with VSV G protein or spike protein of SARS-CoV-2, including various variants of concern, in the absence or presence of DG. NH_4_Cl, E-64d, or camostat was used as control. At 24 h post infection, the cells were replaced with fresh medium. At 72 h after infection, cells were lysed with 30 μL/well of cell lysis buffer (Promega, Madison, WI, USA) for 15 min, followed by addition of 60 μL/well of luciferase substrate (Promega). Firefly luciferase activities were measured by luminometry with an EnSpire instrument (Perkin Elmer, Waltham, MA, USA).

In order to investigate whether serine protease TMPRSS2 could rescue the inhibition of DG on the SARS-CoV-2-S-driven entry, 293T-hACE2 cells seeded into white-wall, clear- bottom 96-well plates were transfected with pcDNA3.1(+)-TMPRSS2. Sixteen hours later, cells were incubated with the mixture of pseudotyped SARS-CoV-2 and DG. The medium was changed the following day and at 72 h after infection, and firefly luciferase activities were measured as described above.

To examine whether DG could enhance the inhibitory effect of other SARS-CoV-2 entry inhibitors, 293T-hACE2 cells transiently expressing TMPRSS2 were treated with DG alone or in combination with NH_4_Cl, E-64d, or camostat when infected with pseudotyped SARS-CoV-2. At 72 h after infection, the firefly luciferase activities were measured as described above.

### 4.11. Time-Resolved Fluorescence Resonance Energy Transfer (TR-FRET)-Based SARS-CoV-2 S-RBD-ACE2 Interaction Assay

Interaction of RBD and ACE2 was detected based on a TR-FRET assay by using an Add&Read SARS-CoV-2 S protein RBD/ACE2 Kit (Vazyme, Nanjing, China). Briefly, 4 μL DG, 4 μL of recombinant SARS-CoV-2 RBD fragment with a Tag1, and 4 μL of ACE2 with a Tag2 were added to a white microplate. Next, 4 μL of anti-Tag1 conjugated with donor Eu and 4 μL of anti-Tag2 conjugated with A2 receptor were introduced. Following a 2 h incubation at room temperature, fluorescence energy transfer from Eu to A2 was measured as the ratio of fluorescence intensity at 665 nm to that at 615 nm (excitation at 320 nm). The standard neutralizing antibody was used as a positive control. Vehicle control, RDV, GC-376, and E-64d were also introduced. Any compound/antibody which could block the interaction of RBD and ACE2 could reduce the value of 665/615.

### 4.12. Capillary Electrophoresis (CE)

Interactions between RBD and DG and between hACE2 and DG were analyzed by CE using the Agilent 7100 Capillary Electrophoresis System (Agilent Technologies Inc., Santa Clara, CA, USA). The desired final concentration of recombinant protein (RBD or hACE2) was mixed with DG in an Eppendorf (EP) tube, and placed in a S1000TM Thermal Cycler PCR machine for 10 min in a metal bath at 37 °C for CE analysis. The total length of the capillary was 50.2 cm, and the effective length was 40 cm. UV was detected at 214 nm. Running buffer was 50 mM pH 8.7 boric acid–borax buffer. Injection was 0.5 psi, 5 s. Separation voltage was 20 kV. Separation temperature was 25 °C. Before the injection of each sample, the capillary was washed for 3 min with 0.1 M NaOH, H_2_O, and electrophoresis buffer, successively. The new capillary was activated by rinsing for 30 min with 1 M NaOH, 0.1 M NaOH, and distilled water, successively.

### 4.13. Surface Plasmon Resonance (SPR) Assay

Interactions between RBD or S protein and the compound DG were analyzed by a SPR assay performed on a CM5 chip integrated into the Reichert4 SPR system (Reichert, Buffalo, NY, USA). Recombinant RBD proteins of SARS-CoV-2 (Wuhan-Hu-1/BA.2) and spike proteins of HCoV-OC43/HCoV-229E were purchased from Sino Biological. All solutions were prepared using ultrapure water obtained from the Master Touch-S15UVF Pure Water Purification system. The running buffer used throughout the analysis was 1% DMSO PBST (pH 7.6 PBS buffer, 0.05% Tween-20, 1% DMSO), which was filtered through a 0.22 µm membrane filter before use. The proteins were prepared with pH 4.5 sodium acetate solution at 200 μL, 0.25 μg/μL. DG was configured with 1% DMSO PBST to the desired gradient concentration. The analysis temperature was controlled at a level of 25 ± 1 °C. Target/control proteins were immobilized on the surface of the gold film of the chip containing carboxymethyl glucan by covalent amine coupling. Target/control proteins were immobilized on the sensing channels, and the reference channel was employed as a negative control. Furthermore, ethanolamine was used as a blocker to fill in the blanks. The final signal from the sensing channel was normalized by subtracting the signal from the reference channel. Target/control proteins were injected into their respective sensing channels at a rate of 10 µL/min for 300 s, and the CM5 chip was then ready for the immobilization of protein. Then, compound DG was injected successively on a concentration gradient into the SPR channels at a speed of 25 µL/min to collect the real-time SPR signal. The binding kinetics and affinity were analyzed using TraceDrawer software (1.6.1, Ridgeview Instruments, Uppsala, Sweden).

### 4.14. Molecular Docking

Computer simulation and molecular docking were used to characterize and verify the binding effect and the sites of DG and RBD complexes. Using this method, we explored all possible binding modes through a fast Fourier Transform (FFT) search strategy, followed by evaluating these binding modes using either the ITScorePP or ITScorePR scoring function. Ultimately, the top-ten best binding modes were selected based on binding energy scores. During this process, the following default settings were employed: a grid spacing of 1.2 Å for 3D searches, and an angular sampling interval of 15 for rotation, with automatic utilization of PDB interface information for model construction. The docking results were analyzed using Protein–Ligand Interaction Profiler [41] (https://plip-tool.biotec.tu-dresden.de/plip-web/plip/index, accessed on 18 June 2023), which can identify non-covalent interactions between biomacromolecules and their ligands, including hydrogen bonds, hydrophobic interactions, salt bridges, π-π stacking, etc. It provides atomic-level binding information along with visualizable and parsable output files. All code is available on GitHub. Three-dimensional structures of the following proteins were obtained from the PDB database: SARS-CoV-2 Wuhan-hu-1 Spike-RBD (PDB: 8DV1); SARS-CoV-2 P.1 Spike-RBD (PDB: 7V7A); SARS-CoV-2 B.1.617 Spike-RBD (PDB: 7OR9); SARS-CoV-2 B.1.351 Spike-RBD (PDB: 7V76); and SARS-CoV-2 BA.2 Spike-RBD (PDB: 7Y75). DG was simulated to translate and rotate around the chemical bonds in the fixed RBDs for docking. False-positive complexes were excluded by short-range electrostatic energy, and unqualified complex configurations were excluded by surface complementarity and geometric fitting. Finally, docking-complex models with different positions were obtained. The most favorable interaction between DG and RBDs is the formation of complexes with the lowest binding energy (the most thermally stable).

### 4.15. Animal Experiments

Pregnant Balb/c mice (16 days) were purchased from SiPeiFu Biotechnology Co., Ltd. (Beijing, China). Mice were housed in an ABSL-2 animal facility under specific pathogen-free conditions. All animal experiments were carried out in accordance with the guidelines of the Animal Care and Welfare Committee of the Institute of Medicinal Biotechnology, Chinese Academy of Medical Sciences and Peking Union Medical College for the Ethics of Animal Care and Treatment (approval number: IMB-20230601-D_11_-01). To test the effect of DG on mortality and morbidity under the HCoV-OC43 infection, 4-6-day-old suckling Balb/c mice were intranasally administered DG (2.5 mg/kg, 5 mg/kg, or 10 mg/kg) in sterile deionized water simultaneously with the challenge of HCoV-OC43 at 1000 TCID_50_ (50% tissue-culture infectious dose tested in H460 cells). For the viral control group, the same volume of sterile deionized water was administered intranasally. Survival rates, body weight changes, and clinical scores were monitored for 11 days (n = 10). The severity of clinical symptoms was scored as follows: 0, healthy; 1, abnormal behavior, social distancing, weight loss; 2, hair scattered, limb shaking, weakness; 3, upper- or hind-limb paralysis, arched back, ataxia, or drowsiness; and 4, moribund or dead.

To test the effect of DG on viral propagation and histopathology in the HCoV-OC43-infected mice, 6-day-old suckling Balb/c mice were intranasally administered DG (10 mg/kg) in sterile deionized water 0.5 h before (−0.5 h) or simultaneously with (0 h) the challenge of HCoV-OC43 (1000 TCID_50_). The mice were euthanized on day 6 after infection (n = 8); viral RNA levels in mouse brain were then determined by qRT-PCR assay. Mice brain tissues were fixed in 10% neutral buffered formalin and applied pathologically for hematoxylin and eosin (H&E) assay and immunohistochemical (IHC) analyses with antibody against HCoV-OC43 N protein (Millipore).

### 4.16. Statistical Analyses

Statistical analyses were performed using GraphPad Prism software (Versions 7 and 9). Results were expressed as mean ± SD. Data were analyzed by one-way ANOVA with Holm–Sidak’s multiple comparisons test or unpaired two-tailed Student’s *t*-test (comparisons between two groups). *p* < 0.05 was considered significant.

## 5. Conclusions

In conclusion, we demonstrated that DG binds to the spike protein of human coronaviruses to block viral entry, and validated DG as a potential broad-spectrum antiviral therapeutic against human coronavirus.

## Data Availability

The data associated with this paper are available upon request to the corresponding author.

## Supplementary Materials

The following supporting information can be downloaded at: https://www.mdpi.com/article/10.3390/ijms26136334/s1.
