# Supplementary material for: Diammonium Glycyrrhizinate Exerts Broad-Spectrum Antiviral Activity Against Human Coronaviruses by Interrupting Spike-Mediated Cellular Entry
Source: Int J Mol Sci. 2025 Jun 30;26(13):6334. doi: 10.3390/ijms26136334 (PMC12250084; doi:10.3390/ijms26136334)
Supplement: Supplementary file 1 [file ijms-26-06334-s001.zip › ijms-3684047-supplementary.pdf]

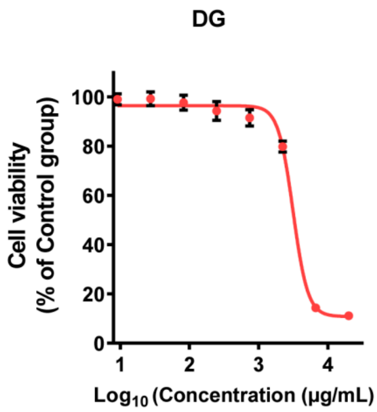

Figure S1. Cytotoxic effect of the DG on 293T-hACE2 cells were determined by a CellTiter-Fluo Cell Viability Assay.

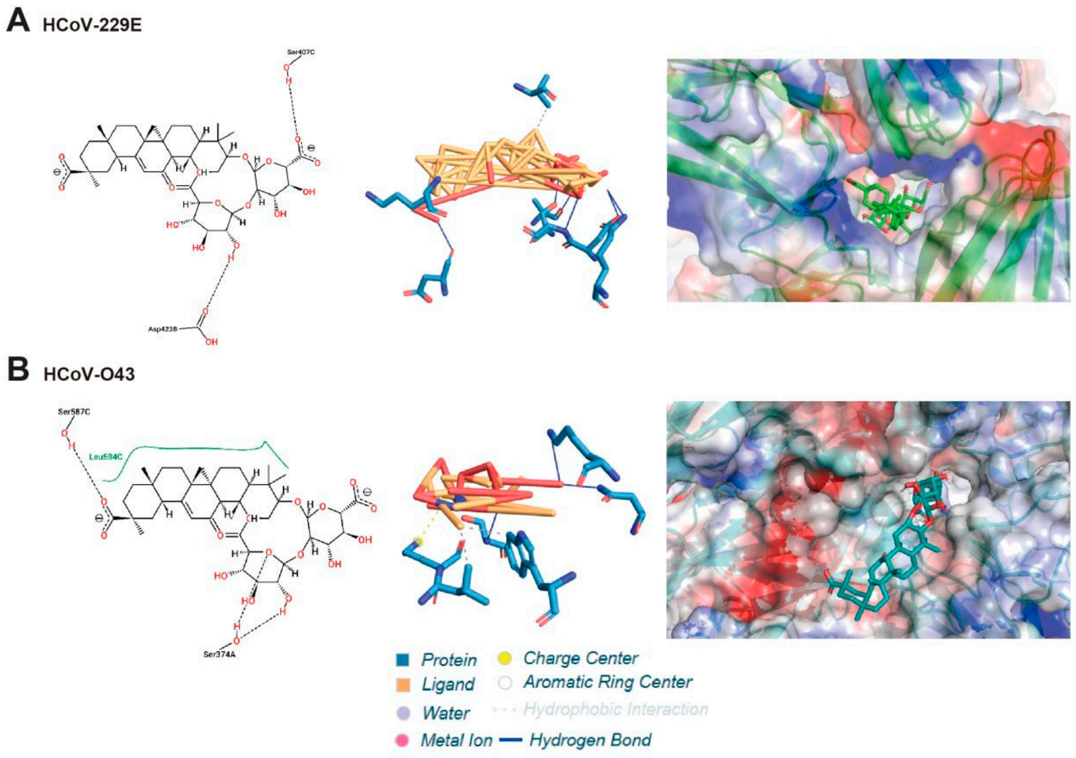

Figure S2. Two-dimensional structure of diammonium glycyrrhizinate and three-dimensional illustration of its interaction with the APN binding pocket of HCoV-229E RBD (A) or with the N-acetyl-9-O-acetylneuraminic acid binding pocket of HCoV-OC43 RBD (B).

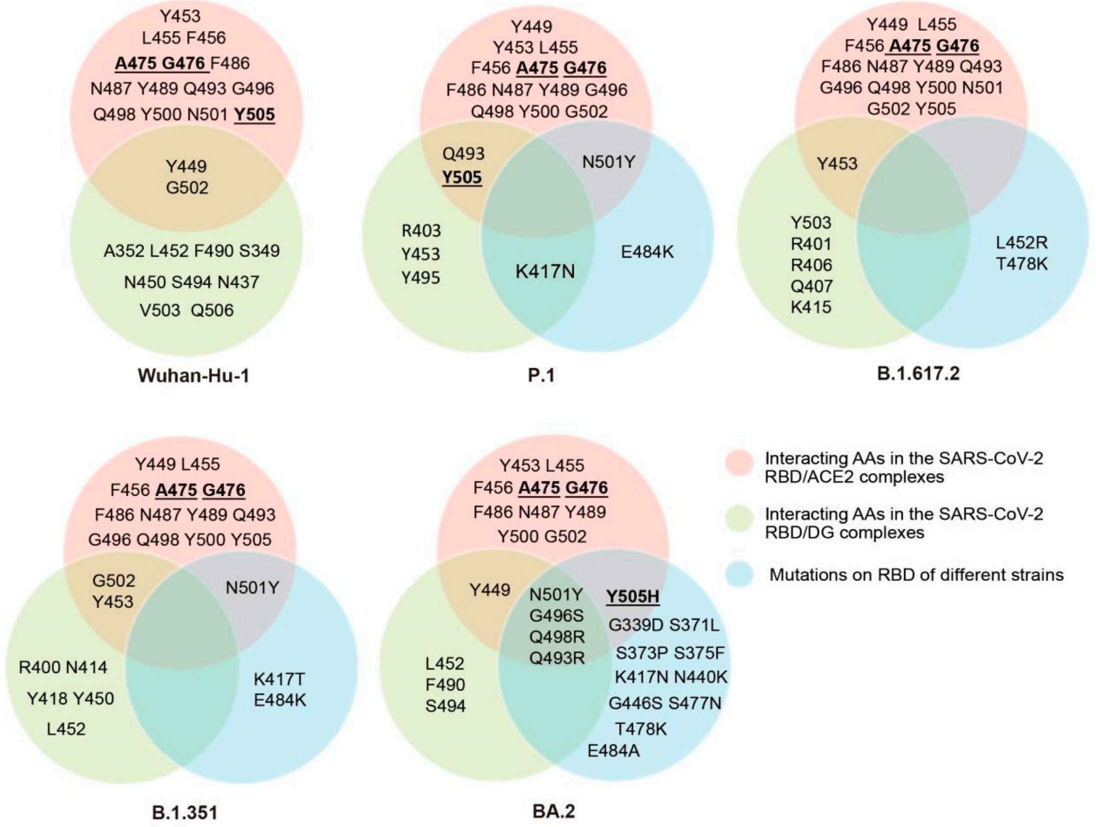

Figure S3. Cross-interacting amino acids (AAs) in SARS-CoV-2 RBD/ACE2 complexes, RBD/DG complexes, and mutations on RBD of different strains.

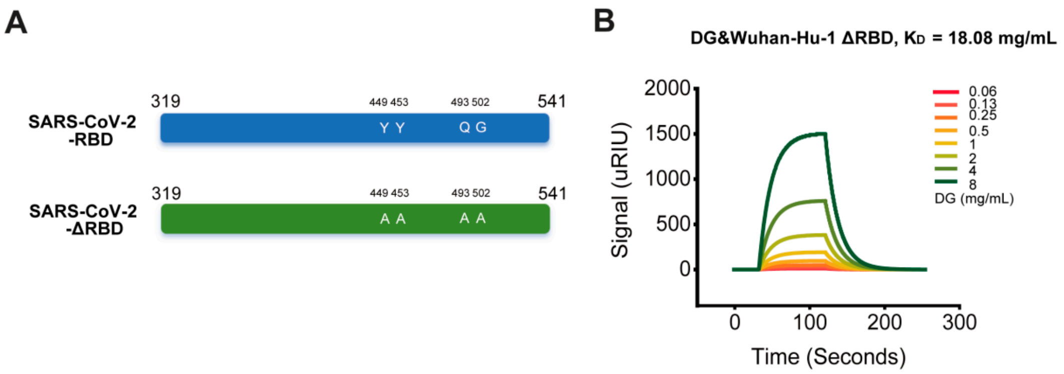

Figure S4. SPR analysis of the interaction between DG with RBD of SARS-CoV-2 (Wuhan-Hu-1) containing four amino acid mutations (Y449A, Y453A, Q493A and G502A). (A) Schematic representation of SARS-CoV-2 RBD protein (319 – 541 aa) and mutated RBD protein. (B) SPR analysis.

Table S1. Interacting residues in the SARS-CoV-2 Wuhan-Hu-1 RBD/DG complexes

Interaction force type	Residue	AA	Distance	Ligand Atom	Protein Atom
Hydrophobic Interactions	352	ALA	3.65	1947	179
	449	TYR	3.88	1943	1117
	452	LEU	3.69	1905	1156
	490	PHE	3.84	1901	1542

Interaction force type	Residue AA	Distance H-A	Distance D-A	Donor Angle	Protein donor	Side chain	Donor Atom	Acceptor Atom
Hydrogen Bonds	349SER	2.01	2.89	142.56	√	X	145 [Nam]	1948 [O3]
	349SER	3.46	3.76	101.02	√	√	150 [O3]	1949 [O3]
	450ASN	2.74	3.09	100.57	√	√	1130 [Nam]	1953 [O3]
	494SER	3.65	4.04	107.46	√	√	1577 [O3]	1944 [O3]

Table S2. Interacting residues in the SARS-CoV-2 B.1.351 RBD/DG complexes

Interaction force type	Residue	AA	Distance	Ligand Atom	Protein Atom
Hydrophobic Interactions	418	TYR	3.78	1950	823
	452	LEU	3.47	1925	1166
	452	LEU	3.63	1916	1166
	453	TYR	3.67	1912	1177
	502	GLY	3.74	1941	1654

Interaction force type	Residue AA	Distance H-A	Distance D-A	Donor Angle	Protein donor	Side chain	Donor Atom	Acceptor Atom
Hydrogen Bonds	400	ARG	2.08	2.82	127.66	√	√	655 [Ng+]
	400	ARG	3.23	3.77	114.33	√	√	658 [Ng+]
	414	ASN	2.07	3.01	152.68	√	√	785 [Nam]
	418	TYR	2.34	3.2	150.17	√	√	825 [O3]
	418	TYR	2.41	2.78	101.72	X	√	1952 [O3]
	450	TYR	2.35	3.27	163.18	√	√	1140 [O3]

	450	TYR	2.66	3.27	121.31	X	√	1958 [O3]
	502	TYR	3.22	3.92	132.81	√	√	1656 [O3]

Table S3. Interacting residues in the SARS-CoV-2 B.1.617 RBD/DG complexes

Interaction force type	Residue	AA	Distance	Ligand Atom	Protein Atom
Hydrophobic Interactions	453	TYR	3.98	1969	1183
	503	TYR	3.99	1965	1669

Interaction force type	Residue AA	Distance H-A	Distance D-A	Donor Angle	Protein donor	Side chain	Donor Atom	Acceptor Atom
Hydrogen Bonds	401	ARG	3.41	3.87	111.19	√	√	664 [Ng+]
	406	ARG	2.63	3.4	131.79	√	√	714 [Ng+]
	407	GLN	2.31	2.97	121.48	√	√	728 [Nam]
	415	LYS	3.06	3.59	113.1	√	x	786 [Nam]

Table S4. Interacting residues in the SARS-CoV-2 P.1 RBD/DG complexes

Interaction force type	Residue	AA	Distance	Ligand Atom	Protein Atom
Hydrophobic Interactions	417	THR	3.67	1909	820
	493	GLN	3.67	1931	1573
	505	TYR	3.77	1944	1686

Interaction force type	Residue AA	Distance H-A	Distance D-A	Donor Angle	Protein donor	Side chain	Donor Atom	Acceptor Atom
Hydrogen Bonds	403	ARG	2.52	3.14	118.93	√	√	693 [Ng+]
	453	TYR	2.41	3.2	141.31	√	√	1174 [O3]
	495	TYR	3.07	3.96	153.68	x	x	1946 [O3]

Table S5. Interacting residues in the SARS-CoV-2 BA.2 RBD/DG complexes

Interaction force type	Residue	AA	Distance	Ligand Atom	Protein Atom
Hydrophobic Interactions	452	LEU	3.66	1959	1157
	490	PHE	3.78	1916	1545
	493	ARG	3.41	1946	1569

Interaction force type	Residue AA	Distance H-A	Distance D-A	Donor Angle	Protein donor	Side chain	Donor Atom	Acceptor Atom
Hydrogen Bonds	449	TYR	2.14	3.05	158.13	√	√	1122 [O3]
	494	SER	1.93	2.87	152.03	√	X	1581 [Nam]
	494	SER	3.2	3.76	118.61	x	X	1969 [O3]
	496	GLY	2.37	3.11	128.09	√	x	1603 [Nam]
	498	ARG	2.91	3.49	116.73	√	√	1630 [Ng+]
	501	TYR	3.58	4.09	116.18	√	√	1664 [O3]

Table S6. Interacting residues in the HCoV-229E RBD/DG complexes

Interaction force type	Residue	AA	Distance	Ligand Atom	Protein Atom
Hydrophobic Interactions	183	VAL	3.54	28627	19825
	298	LYS	3.86	28608	11644
	298	LYS	3.57	28651	11645

Interaction force type	Residue AA	Distance H-A	Distance D-A	Donor Angle	Protein donor	Side chain	Donor Atom	Acceptor Atom
Hydrogen Bonds	342	ASP	2.49	2.91	106.06	x	x	28653 [O3]
	381	LYS	2.55	3.32	132.72	√	√	12430 [N3+]
	405	ALA	3.09	3.89	140.65	x	x	28657 [O3]
	407	SER	2.5	3.23	133.47	√	√	21931 [O3]
	423	ASP	2.13	2.82	128.93	X	√	28667 [O3]
	424	GLY	2.8	3.68	145.29	√	X	12818 [Nam]

Table S7. Interacting residues in the HCoV-OC43 RBD/DG complexes

Interaction force type	Residue	AA	Distance	Ligand Atom	Protein Atom
Hydrophobic Interactions	80	LEU	3.31	33836	23177
	90	TRP	3.55	33836	23276

Interaction force type	Residue AA	Distanc e H-A	Distance D-A	Donor Angle	Protein donor	Side chain	Donor Atom	Acceptor Atom
Hydrogen Bonds	27	ASN	1.84	2.86	174.65	√	√	22675 [Nam]
	29	LYS	3.31	3.69	103.99	√	√	22697 [N3+]
	31	THR	3.53	3.87	103.91	√	√	22717 [O3]
	81	LYS	2.34	3.24	145.37	√	√	23187 [N3+]
	83	SER	3.06	3.87	137.14	√	x	23197 [Nam]

Interaction force type	Residue	AA	Distance	Protein positive	Ligand Group	Ligand Atoms
Hydrogen Bonds	81	LYS	5.3	√	Carboxylate	33818, 33820
